# Daily image-guided carbon ion stereotactic radiotherapy of hepatocellular carcinoma: paving the way for MR-guided online adaptive treatment

**DOI:** 10.1186/s13014-026-02928-4

**Published:** 2026-10-01

**Authors:** Sebastian Regnery, Rita Pestana, Thomas Mielke, Philipp Hoegen-Saßmannshausen, Hanna Waldsperger, Fabian Weykamp, Friderike K. Longarino, Cedric Beyer, Jakob Liermann, Sebastian Klüter, Jürgen Debus, Julia Bauer, Katharina Seidensaal

**Affiliations:** 1 Department of Radiation Oncology, Heidelberg University Hospital, Heidelberg, Germany; 2 Heidelberg Ion Beam Therapy Center (HIT), Heidelberg University Hospital, Heidelberg, Germany; 3 Heidelberg Institute for Radiation Oncology (HIRO) and National Center for Radiation Research in Oncology (NCRO), Heidelberg, Germany; 4 National Center for Tumor Diseases (NCT), Heidelberg, Germany; 5 Clinical Cooperation Unit Translational Radiation Oncology, German Cancer Research Center (DKFZ), Heidelberg, Germany

**Keywords:** Hepatocellular carcinoma, Liver cancer, Stereotactic body radiotherapy, Image-guided radiotherapy, MR-guided radiotherapy, Carbon ion radiotherapy

## Abstract

**Background:**

We aim to quantify the dosimetric benefits of daily online adaptive (DOA) carbon ion stereotactic radiotherapy (CISR) in hepatocellular carcinoma (HCC).

**Patients and methods:**

13 patients received 4 × 10.5 Gy (RBE-weighted dose) CISR to 14 HCC lesions in challenging locations (*N* = 10: liver dome, *N* = 3: < 1 cm from intestines). All patients underwent daily in-room CT, with forward calculation of the baseline plan. Additionally, four patients were shuttled to a 1.5 Tesla MR scanner in treatment position for MRI simulation. Based on visual review of all data, the physician decided to treat or to perform offline plan adaptation and treat another day. We compare the dosimetry properties of four scenarios per fraction: (1) baseline plan, (2) no adaptation, (3) occasional offline adaptation (OOA), (4) DOA.

**Results:**

Baseline planning target volume (PTV) and internal target volume (ITV) coverage by the prescribed dose (median coverage: PTV: 97%, ITV: 100%) significantly decreased for no adaptation (PTV: 87.6%, *p* = 0.0001; ITV: 97.7%, *p* = 0.007) and OOA (PTV: 87.8%, *p* = 0.0001; ITV: 97.7%, *p* = 0.007), while DOA maintained high PTV coverage (PTV: 95.7%, *p* = 0.17; ITV: 100%, *p* = 0.53). Frequent violations of organs-at-risk (OAR) dose constraints occurred for no adaptation and OOA (both 20/52 fractions, 38.5%). They affected the bowel, heart and esophagus up to 6 cm from the PTV. DOA led to considerably fewer (6/52 fractions, 11.5%) and less intense violations of OAR dose constraints.

**Conclusions:**

Daily CT- and MR-guided CISR is feasible in the liver, and demonstrates frequent violations of initial treatment planning objectives when non-adaptive or offline adaptive approaches are followed. DOA could improve target coverage and OAR protection, while particularities of carbon ions such as overdoses far from the target should be considered.

**Supplementary Information:**

The online version contains supplementary material available at https://doi.org/10.1186/s13014-026-02928-4.

## Background

Hepatocellular carcinomas (HCC) represent the 5th most prevalent cancer disease worldwide and are associated with high mortality [1–3]. Even though HCC mostly occur in low- and middle-income countries, incidences are increasing in Northern America and Europe due to more cases of hepatitis and liver cirrhosis [1–3]. Surgical tumor resection represents the treatment of choice, but is often not feasible for technical or medical reasons. Instead, local ablative treatments including radiofrequency ablation (RFA), microwave ablation (MWA) and transarterial chemoembolization (TACE) have been established. However, they face limitations, especially with larger HCC lesions [3, 4]. In recent years, stereotactic body radiotherapy (SBRT) has emerged as an effective alternative. Retrospective analyses and smaller prospective trials have demonstrated high local tumor control and few side effects, so that SBRT can compete with established approaches [5–10]. In specific, SBRT seems useful in larger tumors, where efficacy of other approaches declines [4, 9].

Carbon ion stereotactic radiotherapy (CISR) offers further promise due to its high biological effectiveness and ability to spare organs at risk (OAR) [11]. Our previous phase I trial of CISR in HCC, PROMETHEUS, indicated excellent local control and minimal side effects [10]. This agrees with previous prospective phase I/II trials from Japan [12]. CISR represents a high precision technique that delivers doses with steep distal and lateral gradients due to a narrow Bragg peak [11, 13]. Moreover, carbon ions offer a high linear energy transfer (LET) and thus high biological efficacy [11, 13]. However, CISR is sensitive to interfractional changes because even moderate anatomical alterations translate to relevant changes of the dose distribution, creating unwanted “cold-spots” in the tumor and “hot-spots” in sensitive OAR [11, 14]. Therefore, treating tumors near intestinal OAR or in the liver dome poses considerable challenges due to bowel movements and changes in the position of the diaphragm [11, 14]. Hence, we have excluded patients with HCC in these challenging locations from the previous PROMETHEUS trial [10].

In the past years, our carbon ion facility was equipped with an in-room computed tomography (CT) scanner and a magnetic resonance imaging (MRI) scanner combined with a shuttle system [15]. Moreover, we can now re-calculate the treatment plan on the current anatomy. Hence, patients with HCC undergo daily imaging with plan re-calculation. Then, the treating physician decides to continue with treatment or to postpone treatment and perform offline plan adaptation in the meantime. These sophisticated advances have encouraged us to treat challenging cases of HCC that we would have excluded from CISR before.

Recent advances in online adaptive proton radiotherapy (RT) suggest promising dosimetric improvements in situations with relevant interfractional change [16, 17] and have led to the first clinical implementation [18]. Moreover, MR-guided online adaptive photon SBRT yields superior target volume coverage and OAR protection when treating tumors in the upper abdomen [19–22]. Our vision is to combine the advantages of carbon ions, online adaptive treatment and MR-guidance, which would yield enormous potential to improve target volume coverage and spare OAR. Therefore, we are developing a daily online adaptive workflow for CISR of liver cancer that includes the above-mentioned MRI shuttle system.

In the following, we will:

(1) report on the clinical feasibility of daily CT- and MRI-guided CISR with occasional offline plan adaptation in challenging HCC cases;

(2) quantify the potential dosimetric benefits of daily online adaptive CISR, based on the workflow currently under development.

## Methods

### Patients

This retrospective analysis included patients with HCC who underwent CISR between December 2021 and June 2024. Typically, patients were referred to our institution through a multidisciplinary tumor board due to ineligibility for surgical intervention or other local ablative treatments such as RFA or TACE. Hence, patients presented with complex clinical and anatomical characteristics.

### Treatment planning

Patients were immobilized in supine position on a vacuum pillow, and a belly press was utilized to minimize breathing motion. For treatment planning, a contrast-enhanced four-dimensional (4D) CT was acquired with eight breathing phases. Additionally, a recent contrast-enhanced liver MRI was mandatory. The gross tumor volume (GTV) was defined based on all available imaging. Then, a margin of 5 mm was added to obtain the clinical target volume (CTV), followed by the creation of an internal target volume (ITV). Finally, a margin of 5 mm (7 mm in beam direction) was applied to create the planning target volume (PTV). A total dose of 42 Gy (biologically-weighted dose) in 4 fractions was prescribed to the PTV in a homogeneous fashion. Thus, the aim was to cover at least 95% of the PTV with 95% of the prescribed dose, while limiting the maximum dose to 104%. OAR dose constraints were based on the QUANTEC guidelines [23] and mostly aligned with Timmermann et al. [24] (typical mandatory constraints: maximum spinal cord dose (D_max_) < 25.6 Gy, heart D_max_ < 37 Gy, esophagus D_max_ < 35.6 Gy, stomach D_max_ < 33.2 Gy, bowel D_max_ < 32 Gy, liver-ITV D_mean_ < 12 Gy, and < 700 cm³ liver volume above 10 Gy). Treatment planning was performed using RayStation^®^ (RaySearch Laboratories AB, Stockholm, Sweden) as treatment planning software (TPS). The dose was delivered via a single lateral beam (12/14, 86%) or two angulated lateral beams (2/14, 14%) using pencil beam scanning. All doses were reported as biological equivalent doses to photon radiotherapy (RT) by adjusting for relative biological effectiveness (RBE) with the Local Effect Model I (LEM-I).

### Treatment delivery

Treatment fractions were administered every other day. Patients were immobilized on the treatment couch with abdominal compression and underwent verification CT imaging using a mobile in-room CT scanner (AIRO^®^ system, Brainlab, Munich, Germany). Afterwards, the treatment couch was repositioned in front of the nozzle. Two-plane radiographs were acquired to verify and, if necessary, adjust patient positioning based on bony landmarks. Simultaneously, rigid registration of the planning CT with the verification CT was conducted in the TPS, also using bony anatomy as a reference. Following this, all target volumes and OAR were rigidly registered onto the verification CT, and the baseline treatment plan was recalculated. Finally, the treating physician visually reviewed all data and decided to proceed with the treatment or to postpone treatment and perform offline plan adaptation in the meantime. For four patients, additional daily MRI imaging in treatment position was included for one or two fractions. In these cases, patients were first transferred to a 1.5 Tesla MRI scanner (MAGNETOM Sola^®^, Siemens Healthineers, Forchheim, Germany) using a shuttle system with a hovercraft transfer mattress (Q-Fix^®^ Symphony patient trolley, CQ Medical, Pennsylvania, USA). They underwent a non-contrast-enhanced liver MRI (T1-weighted VIBE and T2-weighted BLADE, approx. 15 min) and were returned to the treatment room for the above-specified CT-based verification process. The MR-images served as MRI simulation and could be imported into the TPS for enhanced localization of the target volume.

### Analysis of intra- and interfractional changes

All available imaging data were imported into RayStation^®^ version 11B. In two cases, two daily CT scans were missing small portions of the beam entry channel due to insufficient field-of-view, so that these four fractions were excluded from all subsequent analyses. The clinical registrations between the planning CT and daily verification CT were utilized to match all images. Both the baseline plan and the offline adapted plans that were used in actual treatment were re-calculated on the daily verification CTs. The GTV was manually edited on the verification CT. Given the limited lesion-to-liver contrast in many instances, two independent methods were employed for comparison: (1) deformable image registration (DIR) using RayStation’s built-in functionality (ANACONDA [25]) was applied between the CT image sets to generate a vector field that enabled consecutive automatic GTV registration (2). MR-informed manual GTV adjustment was applied in cases where daily MRI was available to leverage its superior soft-tissue contrast. The ITV and PTV were automatically transferred from the planning CT to the verification CT using the vector field between the planning and verification (manually edited) GTVs via the above-described DIR algorithm. Additionally, OAR contours were reviewed. Thus, dose volume histograms (DVH) became available to characterize the baseline plan and potentially offline-adapted plans on the daily anatomy. Finally, the baseline plan was re-optimized on the daily anatomy with the initial planning objectives. In six cases, re-optimization was performed both manually and via a fully automated plan optimization tool. This automated plan optimization tool is based on the “Create adapted plan function” available in RayStation^®^ version 11B. Thus, a new plan is created on the daily CT with the same beam and optimization settings as in the baseline plan. Initially, the tool performs 25 optimization iterations, followed by another 25 iterations with robust optimization on the ITV (5 mm isotropic uncertainty). A final optimization filters the beam’s spots and energy layers. Finally, the dose is scaled to fulfill the primary prescription (median dose (D50%) of 42 Gy (RBE) on the ITV). Given the high concordance between the manual and fully-automated approaches (Supplementary Fig. 1), the automated tool was subsequently applied to all cases.

Essential DVH parameters were compared across four treatment scenarios:

1. **Baseline plan**: The initially intended treatment plan.
2. **No adaptation**: Application of the baseline plan across all fractions.
3. **Occasional offline adaptation (OOA)**: Occasional offline adaptations of the baseline plan could be triggered by the physician’s visual review of the current fraction images and applied in the next fraction (current clinical approach).
4. **Daily online adaptation (DOA)**: Daily re-optimization of the treatment plan with the patient on the treatment couch using the fully automated tool (hypothetical).

### Statistics

DVH parameters were compared across the four treatment scenarios using descriptive statistics. Additionally, paired two-sided Wilcoxon signed-rank tests were conducted to assess potential differences between DVH parameters of the baseline plan (scenario 1) and the median values over four fractions in the remaining scenarios. p-values < 0.05 were considered statistically significant, although all findings should be considered exploratory. To accurately estimate differences in PTV coverage across the four scenarios, a Bayesian linear multilevel model (LMM) was applied. Observations were clustered within the 14 irradiated lesions, meaning that observations from different fractions applied to the same lesion were not independent. Thus, we chose a Bayesian approach that is feasible for any number of observations and data structure. The LMM assessed the correlation between the PTV coverage and the respective treatment scenario (population level effect) based on weakly informative priors for the intercept (~ Normal(80, 20)) and treatment scenario (~ Normal(0, 20)). Moreover, two group-level effects were incorporated as varying intercepts, with default flat priors for the standard deviations (half Student-t prior with three degrees of freedom and a scale parameter ≥ 10) [26].

$$\:{PTV}_{coverage}\:\sim\:Normal\left({R}_{Mean},\:{R}_{STD}\right)$$

$$\begin{aligned}\:{R}_{Mean}=intercept&+s\bullet\:scenario+\:\alpha\:\bullet\:I{D}_{lesion}\cr+\beta\:\bullet\:I{D}_{fraction}\end{aligned}$$

*R*_*Mean*_, *R*_*STD*_: *parameters to fit the mean and standard deviation of the assumed normal distribution*.

*s: parameter of population-level effect*.

*ID*_*lesion*_, *ID*_*fraction*_: *identifiers (design matrices) for the irradiated lesion and fraction*.

*α*,* β: parameter vectors of group-level effects*.

Posterior distributions were derived using Markov Chain Monte Carlo (MCMC) simulations, employing 4 chains with 8000 iterations each. Convergence of the chains was verified through R^-values, all of which were ≤ 1.01. To ensure the robustness of the results, sensitivity analyses confirmed that the findings remained consistent under reasonable variations of the prior distributions. Median follow-up was calculated using the inverse Kaplan-Meier approach, while overall survival (OS), progression-free survival (PFS) and local tumor control (LC) rates were estimated using the Kaplan-Meier method. All statistical analyses were conducted using Python version 3.12, with the Bayesian LMM calculations performed in R version 4.4.1 using the brms library.

## Results

### Patient and treatment characteristics

We treated 13 patients with CISR to 14 HCC lesions because one patient was treated twice within one year for a new HCC lesion. Patient characteristics are given in Table 1. Briefly, median age was 69 years [IQR 67–77 years]. Most patients were male (9 / 13 patients, 69%) and presented with advanced liver cirrhosis (Child Pugh Score A – B: 8 patients, 61%). Tumors had a median gross tumor volume (GTV) of 20.5 cm³ [7.7–70 cm³]. Most lesions had previously been treated with at least one local treatment (surgery or local ablative therapy: 8/14 cases, 57%) and were located in a challenging anatomy. Particularly, ten lesion were in the right liver dome (segments IVa, VII or VIII), with two lesions lying < 1 cm from the pericardium, while three lesions were located < 1 cm from the bowel (exclusion criterion in our previous PROMETHEUS trial [10]). In three lesions (21%), the treating physician triggered one offline plan adaptation after two fractions. Two cases (14%) included one daily MRI and another two cases (14%) included two daily MRI acquisitions.

**Table 1:** Patient and lesion characteristics

Patient characteristics (*N* = 13)	
	Median (Q1 – Q3)
**Age [yrs]**	69 (67–77)
**KPI [%]**	90 (70–100)
	**N (%)**
**Sex**	
Female	4 (21%)
Male	9 (69%)
**Child Pugh Score**	
0	5 (38%)
A	6 (46%)
B	2 (15%)
**BCLC Stage**	
A4	6 (46%)
B	3 (23%)
C	4 (31%)
Lesion characteristics (*N* = 14)	
	**Median (Q1 – Q3)**
**GTV [cm³]**	20.5 [7.7–69.7]
**PTV [cm³]**	112.2 [65.0–205.4]
	**N (%)**
**Treatment location**	
< 1 cm from Bowel	3 (21%)
Liver dome	10 (71%)
**Previous treatment for same lesion**	
resection	2 (14%)
local ablative (RFA, TACE, MWA)	6 (43%)

KPI: Karnofsky Perfomance Index. GTV: gross tumor volume. PTV: planning target volume. BCLC: Barcelona Clinic Liver Cancer. RFA: radiofrequency ablation, TACE: transarterial chemoembolization, MWA: microwave ablation

### Target volume coverage

**Fig. 1:**
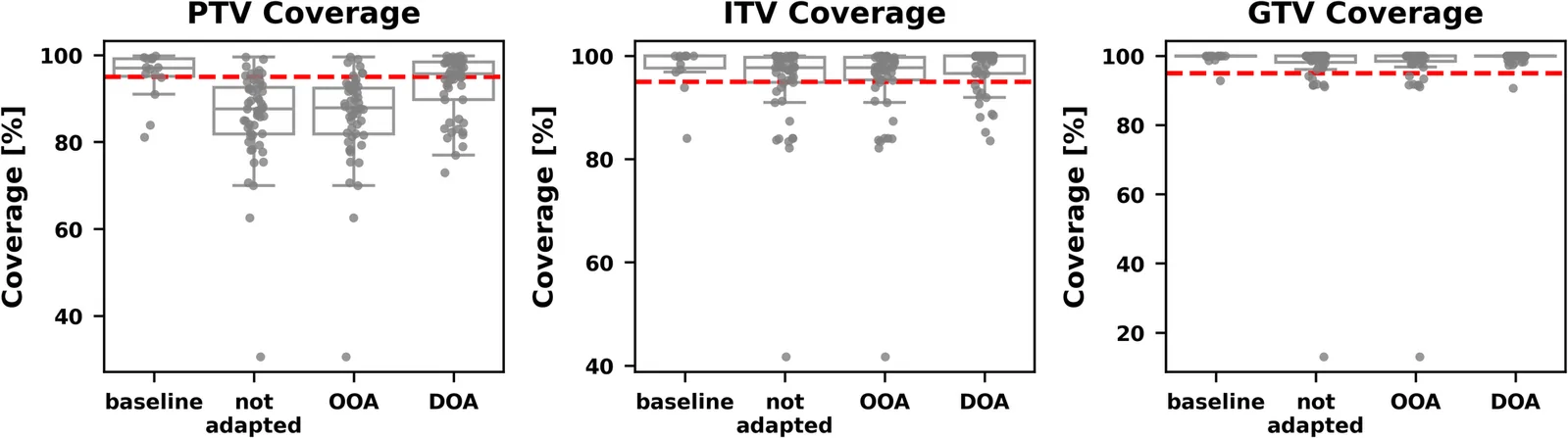
Target volume coverage for different treatment scenarios. The coverage refers to the percentage of the target volume above 95% of the prescribed dose (42 Gy). The red dotted line represents the planning objective that 95% of the planning target volume (PTV, left) should receive at least 95% of the prescribed dose, and is also plotted for the internal target volume (ITV, middle) and gross tumor volume (GTV, right) for better orientation. The baseline plan yields only one value, the other three scenarios contribute 4 fractions per case: no adaptation, occasional offline adaptation (OOA), daily online adaptation (DOA)

We compared the coverage of the target volumes by 95% of the prescribed dose between the four different scenarios: (1) baseline plan, (2) no adaptation, (3) occasional offline adaptation (OOA) triggered by physician’s visual review of the daily images (current clinical approach) and (4) daily online adaptation (DOA, hypothetical). Results are summarized in Fig. 1; Table 2.

**Table 2:** Comparison of dose-volume parameters for different treatment scenarios

Structure	Baseline	Non-adaptive		Offline adaptive		Online adaptive	
Dose Coverage [%]			*p*-value		*p*-value		*p*-value
PTV	97.0 [95.1–99.1]	87.6 [81.8–92.5]	**0.0001**	87.8 [81.8–92.4]	**0.0001**	95.7 [89.8–98.4]	0.17
Auto-PTV	97.0 [95.1–99.1]	85.6 [78.1–92.0]	**0.0001**	85.7 [78.1–91.8]	**0.0001**	94.8 [91.1–97.6]	**0.0001**
ITV	100.0 [97.6–100.0]	97.7 [94.8–99.7]	**0.007**	97.7 [95.3–99.7]	**0.007**	100.0 [96.6–100.0]	0.53
GTV	100.0 [99.8–100.0]	100.0 [98.1–100.0]	0.12	100.0 [98.4–100.0]	0.09	100.0 [99.9–100.0]	0.89
Auto-GTV	100.0 [99.8–100.0]	99.9 [95.9–100.0]	**0.005**	99.9 [96.0–100.0]	**0.009**	100.0 [99.8–100.0]	0.12
Mean Dose [Gy]							
Liver	8.3 [5.4–9.2]	8.0 [5.2–9.4]	0.9	8.0 [5.2–9.4]	0.95	8.7 [5.1–9.7]	**0.03**
V10Gy [%]							
Lung	7.0 [1.2–10.6]	7.9 [0.9–13.3]	0.07	7.9 [0.9–13.3]	0.07	7.8 [0.7–16.8]	**0.049**
Maximum Dose [Gy]							
Heart	20.0 [2.2–28.4]	16.6 [2.8–38.4]	0.14	15.8 [2.8–37.6]	0.14	15.1 [3.0–30.2]	0.24
Spinal cord	0.0 [0.0–0.4]	0.0 [0.0–0.5]	0.74	0.0 [0.0–0.5]	0.74	0.0 [0.0–0.8]	0.61
Esophagus	6.2 [3.9–12.4]	6.0 [3.8–7.9]	0.79	6.0 [3.8–7.9]	0.95	6.4 [3.7–8.9]	0.24
Stomach	14.3 [9.8–19.4]	12.0 [7.8–16.2]	0.06	11.6 [7.7–16.2]	0.06	10.3 [7.2–16.1]	0.15
Bowel	6.1 [4.3–19.9]	8.7 [2.3–24.8]	0.46	8.7 [2.3–24.8]	0.46	7.6 [3.6–22.1]	0.9

Parameters are described by the median and interquartile range. p-values are given for the comparison of the respective scenario with the baseline based on a paired Wilcoxon signed-rank test. Dose coverage refers to aim of covering as much PTV possible by 95% of the prescribed dose. PTV: planning target volume. Auto-PTV: fully automatic PTV based on GTV by deformable image registration. ITV: internal target volume. GTV: gross tumor volume. Auto-GTV: automatically generated GTV by deformable image registration

The PTV coverage was highest in the baseline plan (median 97.0% IQR [95.1–99.1]) and decreased significantly when no adaptation (87.6% [81.8–92.5], *p* = 0.0001) or OOA were performed (87.8% [81.8–92.4], *p* = 0.0001), while only a small non-significant decrease was observed for DOA (95.7% [89.8–98.4], *p* = 0.17). In total, 71.4% (10/14) of baseline plans covered 95% of the PTV with 95% of the prescribed dose. This aim was reached in 15.4% (8/52), 13.5% (7/52) and 55.8% (29/52) of fractions for no adaptation, OOA and DOA, respectively. The Bayesian linear multilevel model (LMM) was compatible with a baseline PTV coverage of 95% [99% compatibility interval: 88–102%] that decreased by -9% [-15 – -3%] for both no adaptation and OOA, but only by -2% [-8–4] for DOA. Accordingly, DOA would increase the PTV coverage by + 7.3%, [4–10%] compared to no adaptation and by + 7.1%, [4–10%] compared to OOA (Supplementary Figs. 2 and 3).

Similarly, the ITV coverage by 95% of the prescribed dose was highest in the baseline plan (median 100.0% IQR [97.6–100]) and decreased significantly for no adaptation (97.7% [94.8–99.7], *p* = 0.007) and OOA (97.7% [95.3–99.7], *p* = 0.007), while no change was observed for DOA (100% [96.6–100], *p* = 0.53). ITV coverage was above 95% in 85.7% (12/14) of the baseline plans, which decreased to 71.2% (37/52), 75% (39/52) and 78.8% (41/52) of fractions for no adaptation, OOA and DOA, respectively (Fig. 1). The GTV coverage by 95% of the prescribed dose was not significantly different between the scenarios, although we observed a trend towards decreased GTV coverage for no adaptation and OOA (Table 2). Still, GTV coverage was above 95% in 92.9% (13/14) of the baseline plans, compared to 86.5% (45/52), 86.5% (45/52) and 98.1% (51/52) of fractions for no adaptation, OOA and DOA, respectively (Fig. 1).

To account for the limited lesion-to-liver contrast when contouring the GTV on daily in-room CT images, we also investigated (1) fully automatic PTVs based on DIR-generated GTVs and (2) PTVs based on MRI-informed manual GTVs (Supplementary Figs. 4 and 5). Overall, the volumetric Dice similarity coefficients (DSC) and mean distance to agreement (MDA) demonstrated a good agreement of the manual CT-based PTVs with the fully automatic contours (mean DSC.92, mean MDA 1.5 mm) and a fair agreement with the MRI-informed contours (DSC 0.75–0.9, MDA 1.5–3 mm). The underlying manual CT-based GTVs and fully automatic GTVs still demonstrated good agreement (mean DSC 0.83, mean MDA 1.8 mm), whereas comparisons of the manual CT-based GTVs and MR-informed GTVs varied between good and insufficient (DSC 0.62–0.89, MDA 1.4–5 mm). Furthermore, calculation of dose volume parameters led to similar values for fully automatic PTVs (Supplementary Fig. 6), although the decrease of the PTV coverage from the baseline was overall higher and also reached statistical significance for DOA (baseline plan: 97.0% [95.1–99.1]; no adaptation: 85.6 [78.1–92.0], *p* = 0.0001; OOA: 85.7 [78.1–91.8], *p* = 0.0001; DOA: 94.8 [91.1–97.6], *p* = 0.0001). Accordingly, the Bayesian LMM yielded similar results when analyzing the fully automatic PTVs (Supplementary Table 1, Supplementary Figs. 7 and 8). MRI-based PTVs were only available in a limited number of fractions, but suggested even stronger declines of the PTV coverage (Supplementary Fig. 9) especially for no adaptation and OOA (baseline plan: 97.0% [95.1–99.1]; no adaptation and OOA identical: 73.5 [71.1–77.7], *p* = 0.13; DOA: 87.1 [79.7–92.5], *p* = 0.13).

### OAR constraints

**Fig. 2:**
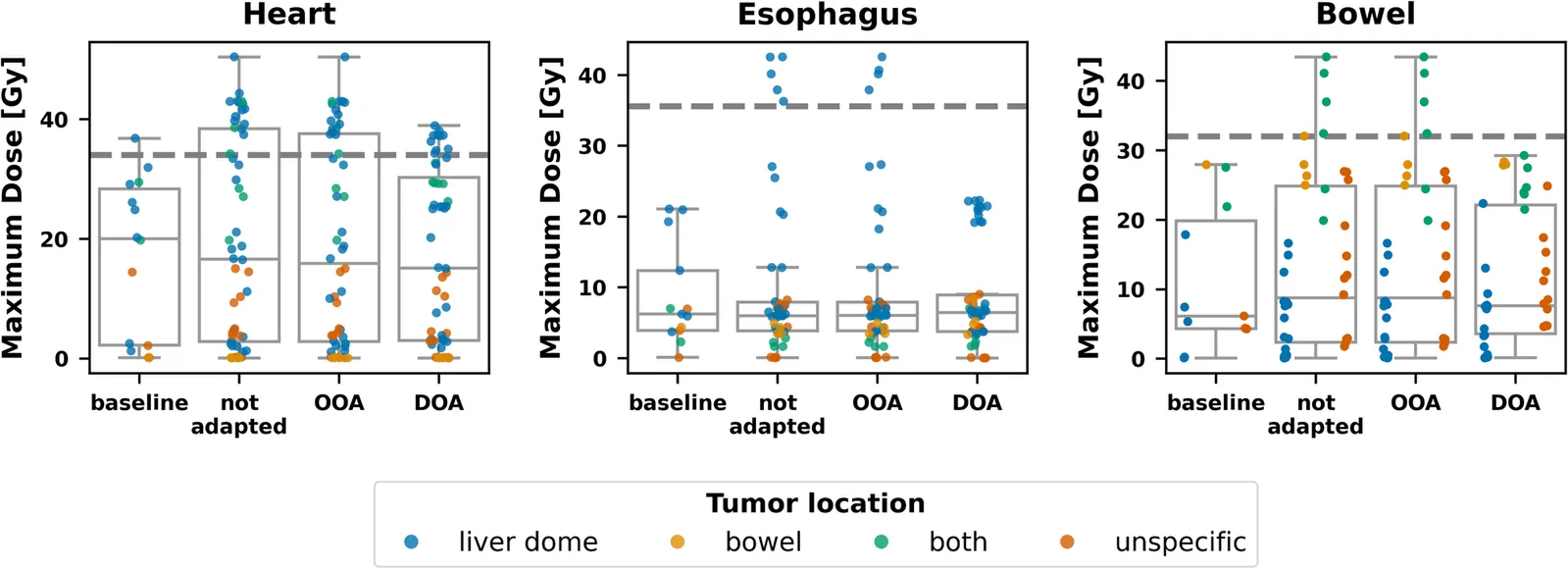
Maximum doses inside different serial organs-at-risk for different scenarios. The gray dotted lines represent the planning dose constraint. The baseline plan yields one value, the other three scenarios contribute 4 fractions per case: no adaptation, occasional offline adaptation (OOA), daily online adaptation (DOA)

**Fig. 3:**
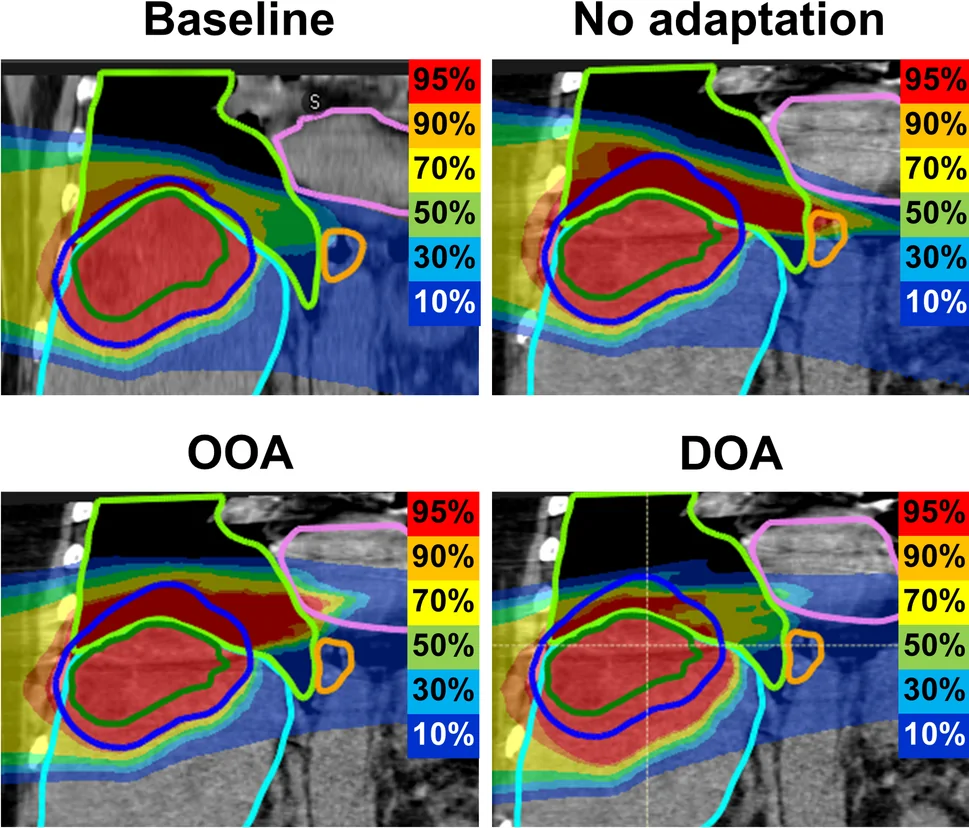
Carbon ion stereotactic radiotherapy of a tumor in the liver dome. Upper left: Baseline plan with excellent planning target volume (PTV: blue) and gross tumor volume (GTV: dark green) coverage and sparing of mediastinal organs (heart: pink, esophagus: orange). Upper right: Simulated non-adaptive scenario using the baseline plan on the daily anatomy. Due to a caudal shift of the liver, the beam “overshoots” into the mediastinum and causes high dose exposition of heart and esophagus. Lower left: In the clinic, occasional offline adaptation (OOA) yielded a plan that had been adapted based on the previous fraction. Despite some improvements, it did not sufficiently spare heart and esophagus. Lower right: Simulated daily online adaptation (DOA) would yield a plan that spares heart and esophagus with moderate compromises to PTV areas inside the lung (light green)

Overall, we could not find any significant difference of maximum doses inside serial OARs (Table 2). Nevertheless, individual violations of dose constraints were similarly frequent when no adaptation or OOA were performed (20 / 52 fractions with ≥ 1 violated constraint, 38.5%). In specific (Fig. 2):

- Violations of bowel dose constraints occurred in three patients (one patient: 1/4 fractions, two patients: 2/4 fractions) where the HCC had close proximity (< 1 cm) to a bowel segment.
- Violations of esophagus dose constraints were observed in two patients (1/4 fractions and 3 (OOA) – 4 (not adapted) /4 fractions) where the HCC was located in the liver dome (Fig. 3).

- Violations of heart dose constraints were observed in seven patients (two patients: 1/4 fractions, one patient: 2/4 fractions, one patient: 3/4 fractions, three patients: 4/4 fractions) where the HCC was located in the liver dome (Fig. 3).

Overdoses in the heart and esophagus occurred due to lower position of the liver dome with consecutive “overshoot” of the particle beam and unintentional mediastinal high dose areas up to 6 cm away from the PTV.

DOA considerably reduced individual dose constraint violations (6 / 52 fractions with 1 violated constraint, 11.5%). Only moderate violations of the heart dose constraints remained in three patients (Fig. 2, two patients: 1/4 fractions, one patient: 4/4 fractions). These violations were deemed clinically acceptable to maintain good PTV coverage. Finally, DOA led to statistically significant albeit moderate increases in the lung V_10Gy_ (baseline: median 7.0% IQR [1.2–10.6], DOA scenario: 7.8% [0.7–16.8], *p* = 0.049) and mean liver dose (baseline: 8.3 Gy [5.4–9.2], DOA scenario: 8.7 Gy [5.1–9.7], *p* = 0.03) compared to the baseline plan (Supplementary Fig. 10**)**.

### Clinical outcomes

After a median follow-up of 15 months, only one lesion (7%) had progressed locally (in-field) after 17 months. Furthermore, three cases (21.4%) of out-field liver progression were observed. Consequently, the OS, PFS and LC rates were 78% (34–83%), 64% (95% CI: 46–92%) and 100% [100–100%] after 1 year. Acute adverse events occurred after eight treatments (57%) and were mostly mild, with six (43%) grade (G) 1 (erythema, fatigue, diarrhea, nausea, elevation of liver enzymes), one (7%) G2 (abdominal pain) and two (14%) G3 (cholangitis, portal vein thrombosis) toxicities. Late adverse events were observed after three treatments (21%), with two (14%) G1 (asymptomatic pleural effusion and rib fracture) and one (7%) G3 (portal vein thrombosis) toxicities. No acute or late G 4–5 toxicities were observed.

## Discussion

### Adaptive particle radiotherapy

Adaptive particle therapy is a developing area in radiation oncology [14, 16–18, 27–31]. Previous studies focus on repeated offline plan re-calculations and potentially adaptations based on weekly re-planning CTs, e.g. in carbon ion RT for lung cancer [32] and proton RT for esophageal and head and neck cancers [28, 31]. Very recently, clinical approaches to online adaptive proton RT have been developed [16, 17] and were translated into clinical practice for cases with rigid anatomical changes such as skull base tumors [18]. At our facility, we can already perform online plan re-calculation so that the treating physician can decide whether to treat or postpone treatment and instead perform (occasional) offline adaptation (OOA). Currently, we are refining fully automated tools for fast online re-optimization. Together with our MR-shuttle system, we possess the essential prerequisites for an MR-guided online adaptive workflow.

A growing body of studies in MR-guided online adaptive photon SBRT demonstrates superior target coverage and sparing of neighboring OAR when treating tumors in the upper abdomen [19–22]. However, (online) adaptive particle treatment comes with especially high workloads and treatment times [30]. So far, only few studies have investigated the potential benefits of adaptive particle SBRT in the upper abdomen. Considering carbon ions, Molinelli et al. [14] compared different robust optimization approaches to a simulated online adaptive CISR of pancreatic cancer based on repeated pre-treatment 4D-CT images. They concluded that only efficient online adaptation strategies can maintain high target coverage in most fractions while sparing the duodenum. Considering HCC, Nenoff et al. [33] have simulated adaptive versus non-adaptive photon and proton SBRT courses in ten patients based on daily setup MR images from a photon MR-linac treatment. They found proton SBRT provided better OAR sparing over photons, but the impact of DOA seemed limited. Conversely, Moteabbed et al. [27] created daily online adaptive MR-guided, offline MR-guided and CBCT-guided proton SBRT plans based on different PTV margins. They demonstrated that transitioning from CBCT-guided to daily online adaptive MR-guided reduced PTV margins, therefore enhancing OAR sparing.

Here, we present the first analysis of original treatment plans and images derived from patients who received CISR to HCC with OOA.

### Target volumes

Interfractional anatomical changes often reduce the PTV coverage, which may be tolerable as long as the PTV is a sufficient “buffer” to maintain coverage of clinical targets. However, we observed that the ITV and, to a lesser degree, the GTV were affected by interfractional changes as well. One important reason for the observed changes appeared to be variable positioning of the belly press. Target volume coverages were maintained by DOA, but not by our OOA workflow. One essential point to consider is that our OOA workflow relies on visual review of re-calculated plans on rigidly registered contours. This may allow fast and pragmatic online evaluation, but could also introduce misinterpretations compared to accurate contour editing. Previous analyses of MR-guided photon SBRT have suggested that visual reviews alone are insufficient to determine the need for online plan adaptation [19]. In specific, subjective visual review of a daily image without editing contours and re-calculation of the baseline plan could not reliably determine when plan adaptation is needed, while a workflow based on re-contouring and re-calculation yielded a sensitivity of 90% [19]. This aligns well with our data because offline adaptation was only triggered once (mid-treatment) in 3/14 (21%) lesions, even though relevant deviations from planning objectives were found in more cases when using re-contouring and re-calculation (25% of fractions failed to provide high ITV coverage, > 50% of fractions with violations of heart dose constraint). Furthermore, one should bear in mind that OOA is quite different from DOA because the plan is re-optimized on past imaging. Thus, OOA mainly aims to increase the general plan robustness, while DOA copes flexibly with day-to-day interfractional changes. All in all, DOA appeared to yield superior target volume coverage. Finally, compromised GTV visibility on non-contrast-enhanced daily CT images represents an important limitation of our analyses and will impede both OOA and DOA in the future. While manual and automatic target volumes agreed well, the comparison between manual CT-only and MR-informed contours yielded good - fair agreements for the PTV but partly insufficient agreements for the GTV. This suggests that future workflows will likely benefit from MRI-guidance to yield highest contour accuracy.

### Organs-at-risk

Similar to our results from target volume coverage, interfractional anatomical changes frequently cause violations of OAR dose constraints. DOA prevented the majority of potential overdoses to OAR, whereas OOA barely seemed beneficial. Several cases of HCC in the liver dome demonstrated dose hotspots in the mediastinum with potential overdoses in the esophagus and heart as far as 3–6 cm from the PTV. These occurred because a lower position of the liver dome opened the way for “beam overshoots” through lung tissue. It is well known that carbon ion dose distributions are not robust near tissue-air interfaces [11]. Still, our results make clear that methods from MR-guided online adaptive photon RT cannot simply be adopted in carbon ion treatment. Particularly, OAR can safely be re-contoured within 3 cm of the PTV to streamline MR-guided online adaptive photon RT [34]. Online adaptive CISR will require more extensive re-contouring, at least in beam direction. We observed small increases of mean doses inside the right lung and liver for DOA. However, these increases did not appear clinically relevant. Slightly elevated lung and liver doses as well as few residual heart dose constraint violations may also reflect the demands of efficient online re-optimization.

### Clinical

Our image-guided CISR workflow with OOA allowed treatment of HCC in difficult locations with good clinical outcomes. After an intermediate median follow-up time of 15 months, local tumor control (in-field) was good. Therefore, the superior target coverage with DOA might not only ensure high long-term local tumor control but could also be exploited to reduce PTV margins. Consequently, doses to OAR could be further decreased, which would lower the risk of side effects in general and prevent severe toxicity in individual patients, such as the case with repeated high dose exposure of the esophagus. In this cohort, we observed moderate rates of adverse events, without any G 4–5 events. The three incidents of G3 toxicity, namely portal vein thromboses and cholangitis, were not necessarily associated with RT but could also be a consequence of the underlying liver cirrhosis. Potentially, an even wider spectrum of difficult cases could be managed using DOA.

Currently, the OOA workflow already includes daily CT and potentially MRI, rigid contour registration and forward calculation of the baseline treatment plan onto the daily imaging as well as visual review by the physician. Therefore, the remaining steps towards creating a clinically feasible DOA workflow include re-contouring, plan re-optimization and quality assurance (QA). Firstly, re-contouring is usually performed manually and remains the most time-consuming step in current MR-guided online adaptive photon RT, with approximately 10–20 min in the upper abdomen [34–36]. These times could further increase because re-contouring within 3 cm of the PTV might not be sufficient in CISR. Therefore, new developments in AI-based contouring should be implemented to reduce the contouring times [37, 38]. Secondly, our automated plan optimization tool in RayStation^®^ could already be implemented into a clinical workflow, managing a full plan re-optimization within mean 2–3 min. Finally, a clinically robust QA procedure still needs to be set up. Based on our experiences with QA for adapted proton plans as well as previous data from online adaptive MR-guided photon RT [35], it seems realistic to implement a streamlined QA workflow that requires up to 10 min.

### Limitations and outlook

Even though our work supports the benefits of DOA in carbon ion irradiation of liver cancer, further developments, especially in imaging and re-contouring, are needed to enable full clinical application. Generating consistent GTVs was difficult from non-contrast enhanced daily CT images, which limits our analyses of target volume coverage as well as implementation of future CT-based DOA workflows. Although we found good agreement between manual and automatic contouring approaches, the comparison between CT and MR-based contours suggested potentially relevant differences. Implementing daily MRI could overcome these limitations in a transition to MR-guided DOA. However, the integration of MRI and DOA will increase treatment time and still requires significant technical refinements. Future approaches towards in-room MR-guided treatment may allow MR-based patient positioning and beam gating, which would further reduce interfractional changes and intrafractional changes.

## Conclusions

Daily CT- and MR-guided CISR is feasible in the liver. While OOA based on visual review of online re-calculated plans demonstrated few benefits, DOA could improve target volume coverage and sparing of OAR. Future DOA implementations should consider particularities of carbon ion treatment, such as possible overdoses far from the target.

## Supplementary Information

Below is the link to the electronic supplementary material.

Supplementary Material 1

## Data Availability

All data underlying this manuscript may be shared anonymously and in accordance with the institutional review board regulations on reasonable request to the authors.

## Abbreviations

CISR: Carbon ion stereotactic radiotherapy
CT: Computed tomography
CTV: Clinical target volume
DIR: Deformable image registration
DOA: Daily online adaptation
DSC: Dice similarity coefficients
GTV: Gross tumor volume
HCC: Hepatocellular carcinoma
ITV: Internal target volume
LC: Local control
LMML: inear mixed model
MDA: Mean distance to agreement
MWA: Microwave ablation
MR: Magnetic resonance
OAR: Organ at risk
OOA: Occasional offline adaptation
OS: Overall survival
PFS: Progression-free survival
PTVP: lanning target volume
RBE: Relative biological effectiveness
RFA: Radiofrequency ablation
RT: Radiotherapy
SBRT: Stereotactic body radiotherapy
TACE: Transarterial chemoembolization
TPS: Treatment planning system
