# Supplementary material for: Daily image-guided carbon ion stereotactic radiotherapy of hepatocellular carcinoma: paving the way for MR-guided online adaptive treatment
Source: Radiat Oncol. 2026 Oct 1;21:133. doi: 10.1186/s13014-026-02928-4 (PMC13632404; doi:10.1186/s13014-026-02928-4)
Supplement: Supplementary file 1 [file 13014_2026_2928_MOESM1_ESM.pdf]

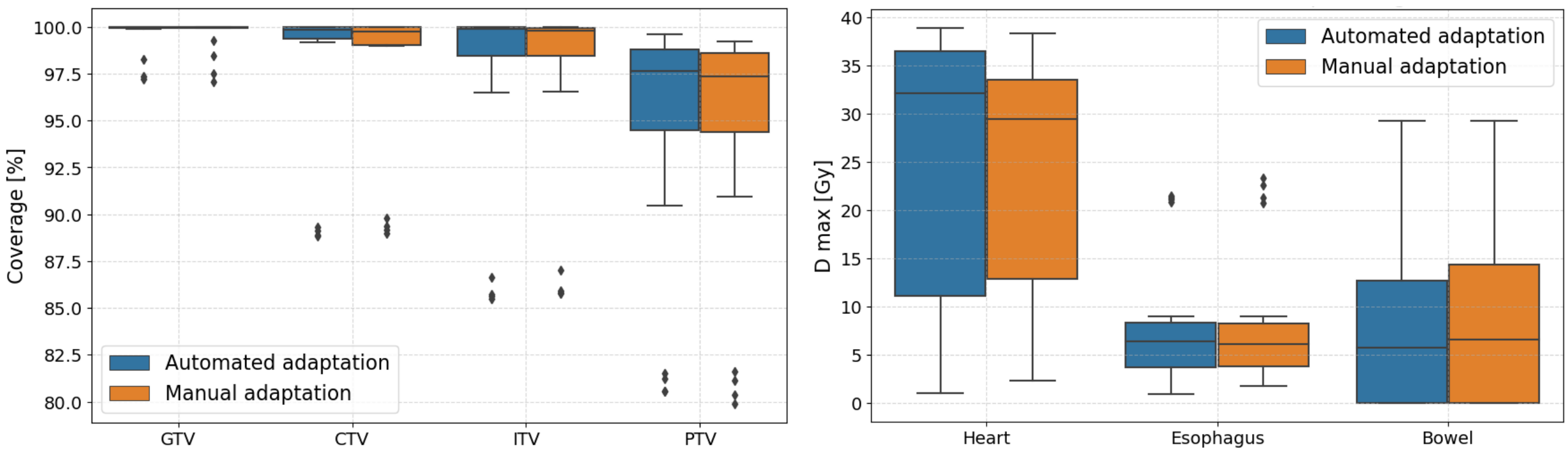

Supplementary Figure 1: Comparison between the automated and manually designed plans for the target volumes and OAR.

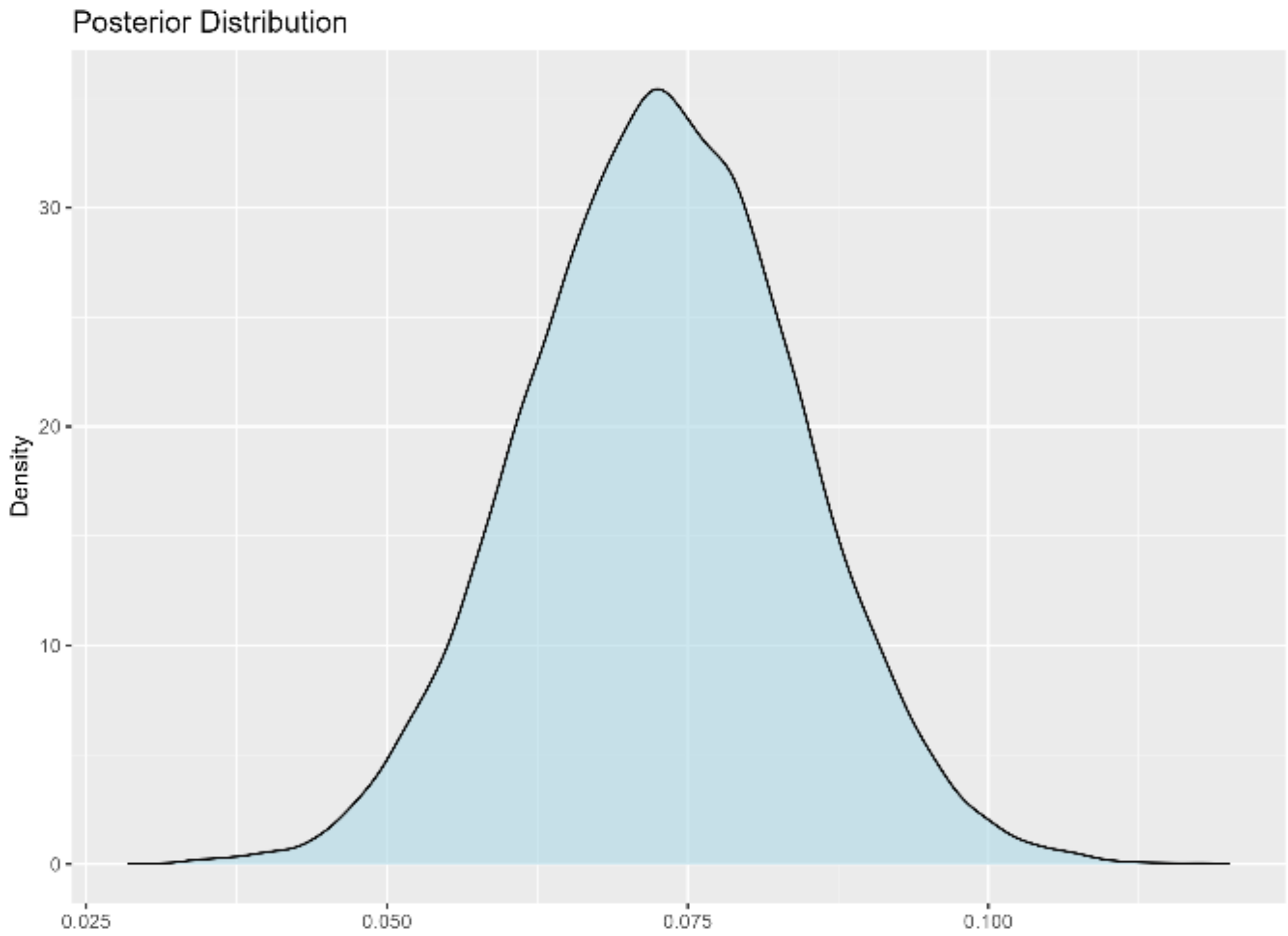

Supplementary Figure 2: Posterior distribution of the planning target volume coverage difference between the daily online adaptive and non-adaptive scenario.

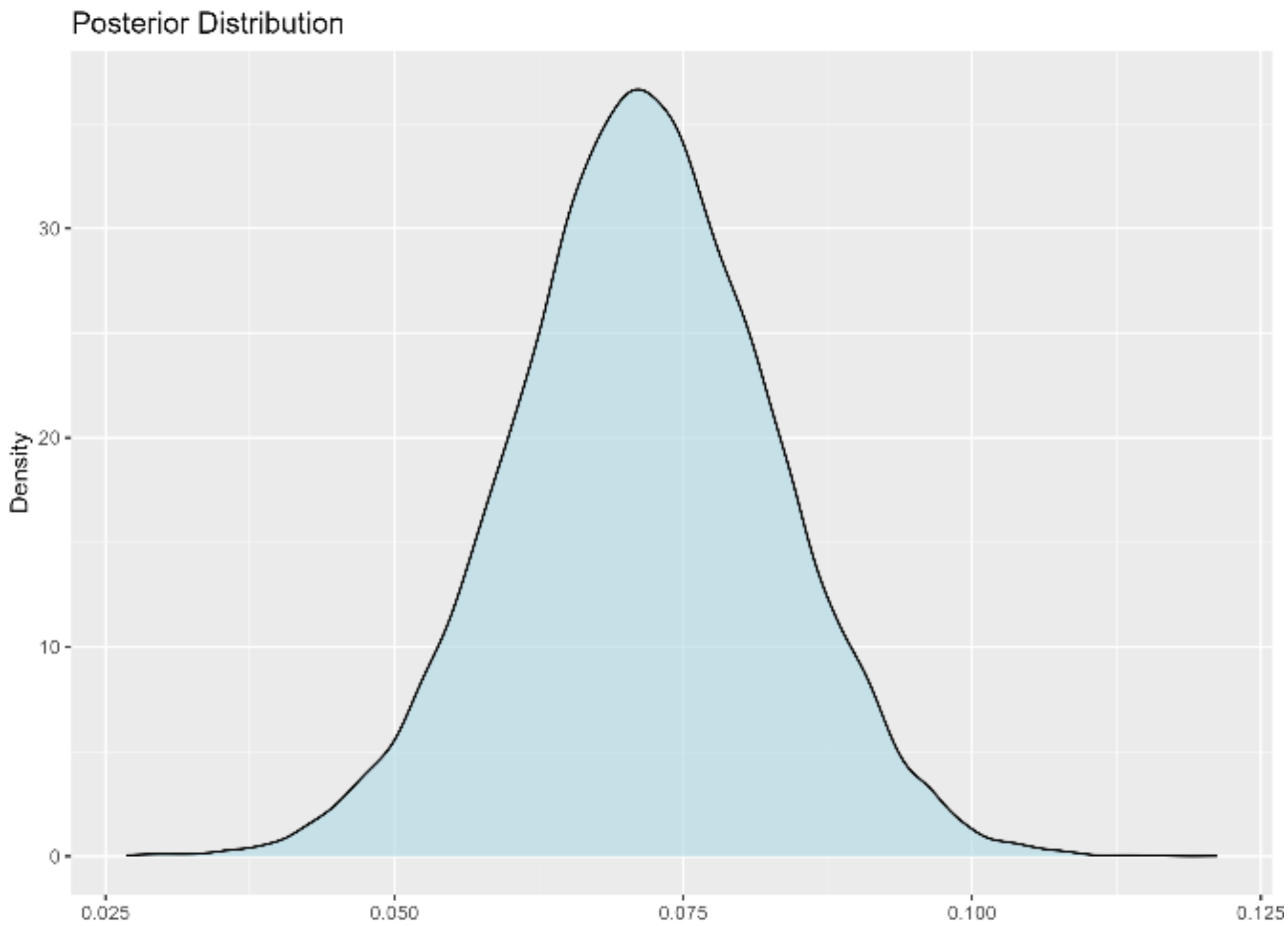

Supplementary Figure 3: Posterior distribution of the planning target volume coverage difference between the daily online adaptive and offline adaptive scenario.

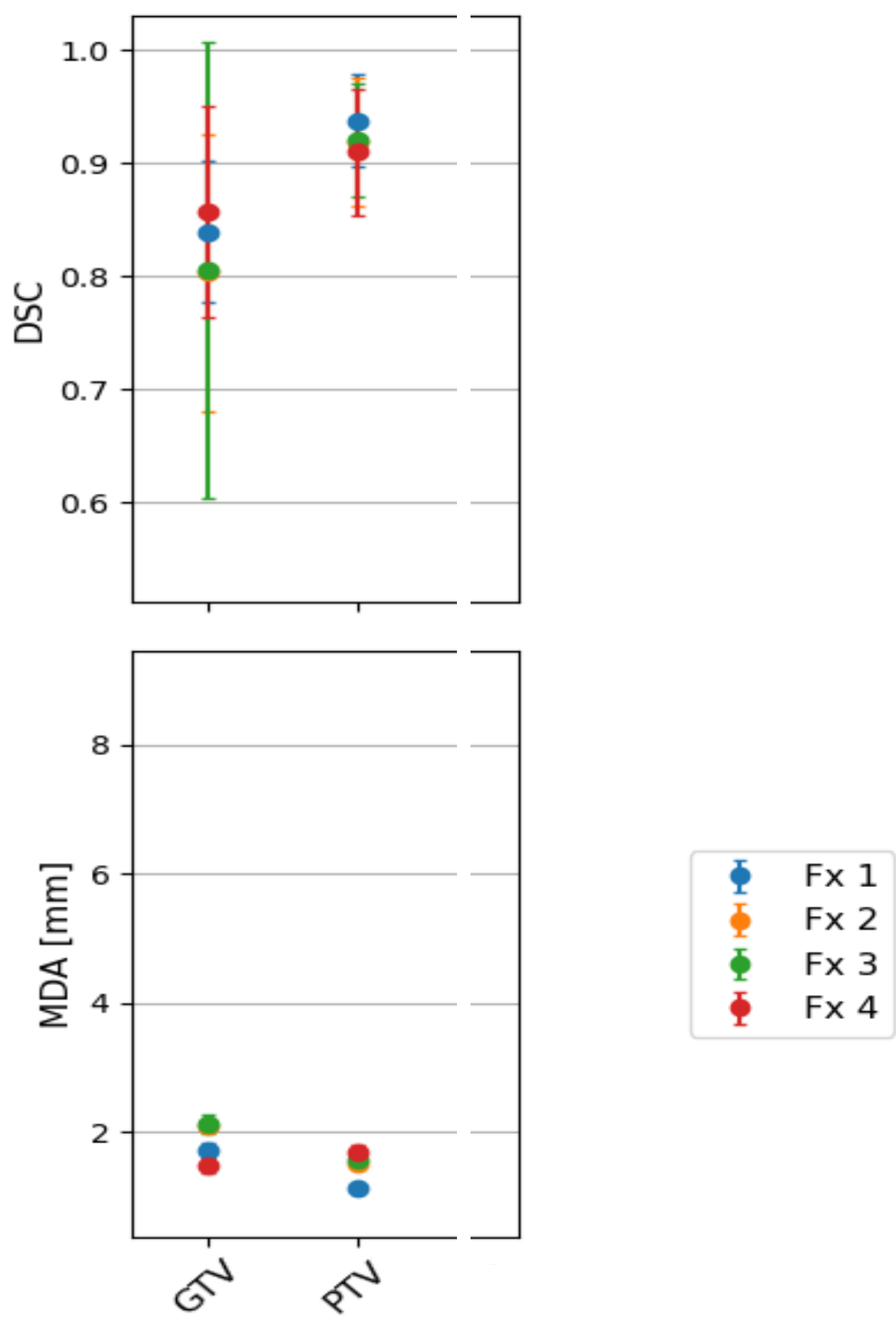

Supplementary Figure 4: Dice similarity coefficient (DSC) and mean distance to agreement (MDA) values obtain from the comparison of the manually contoured structures against those mapped with DIR.

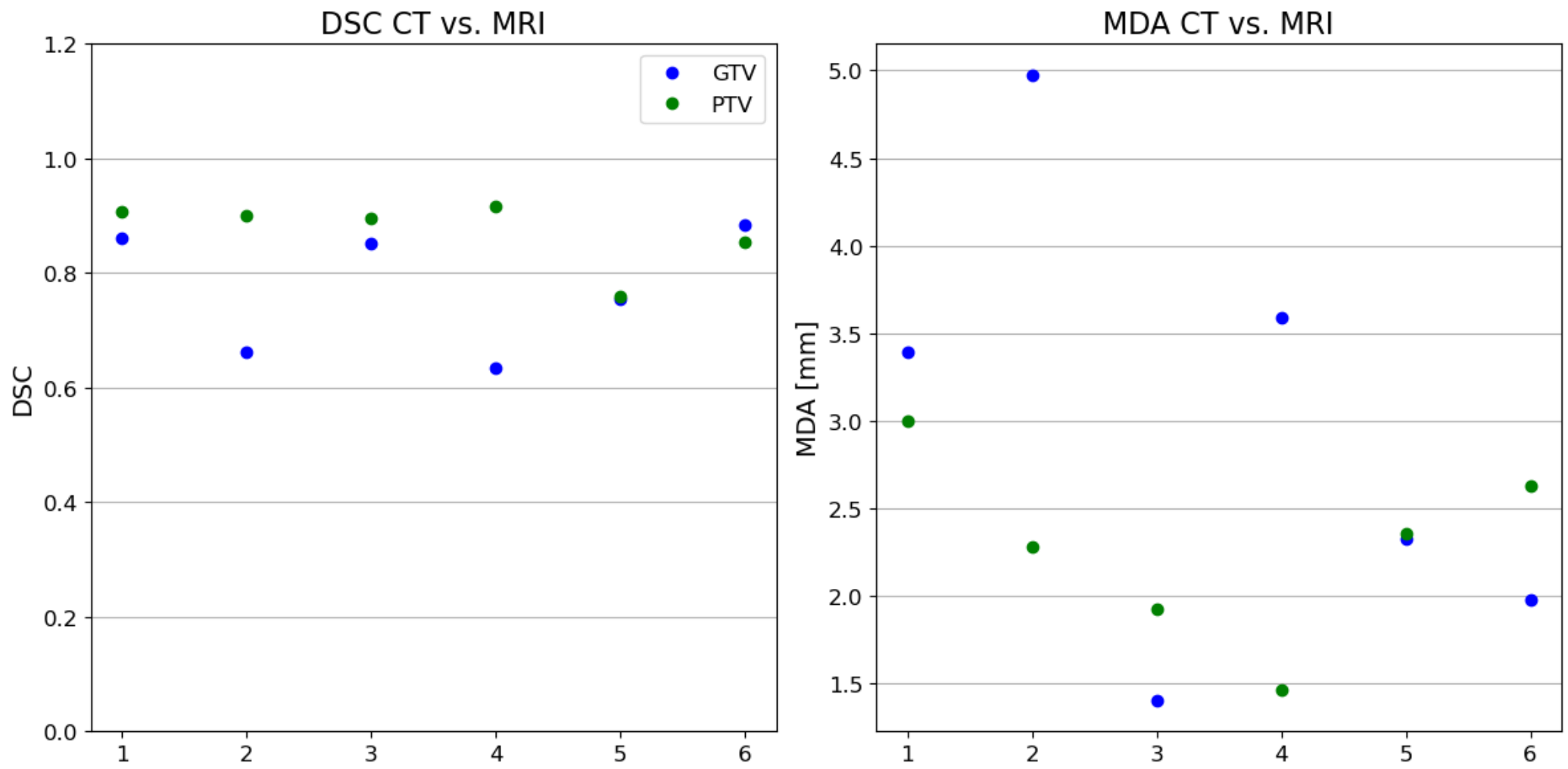

Supplementary Figure 5: Comparison between the CT-based against the MR-based targets. Each number on the x-axis represents one patient.

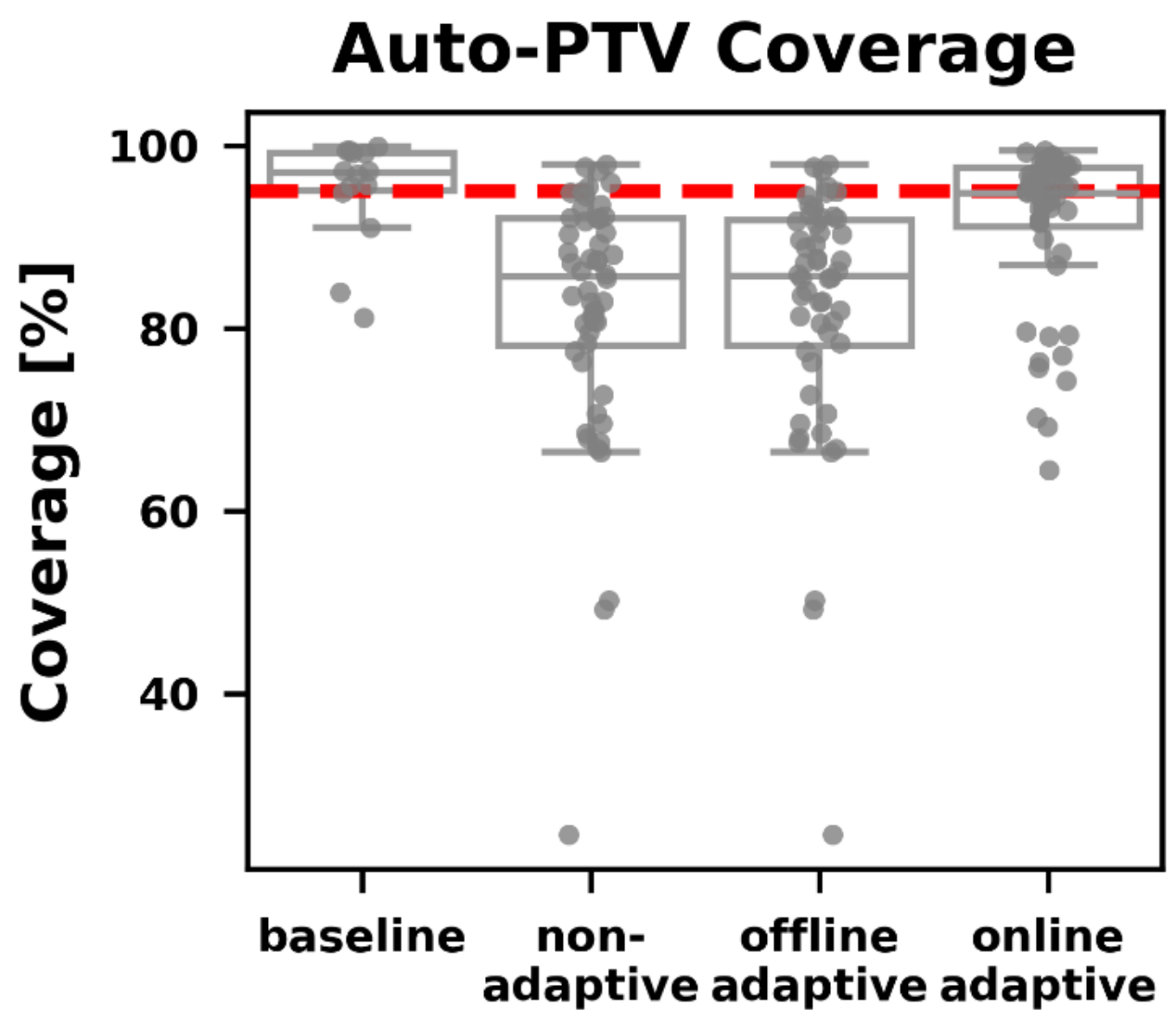

Supplementary Figure 6: Coverage of planning target volume (PTV) based on the automatically generated gross tumor volume (GTV) for the different scenarios. The coverage refers to the percentage of the PTV above 95% of the prescribed dose (42 Gy).

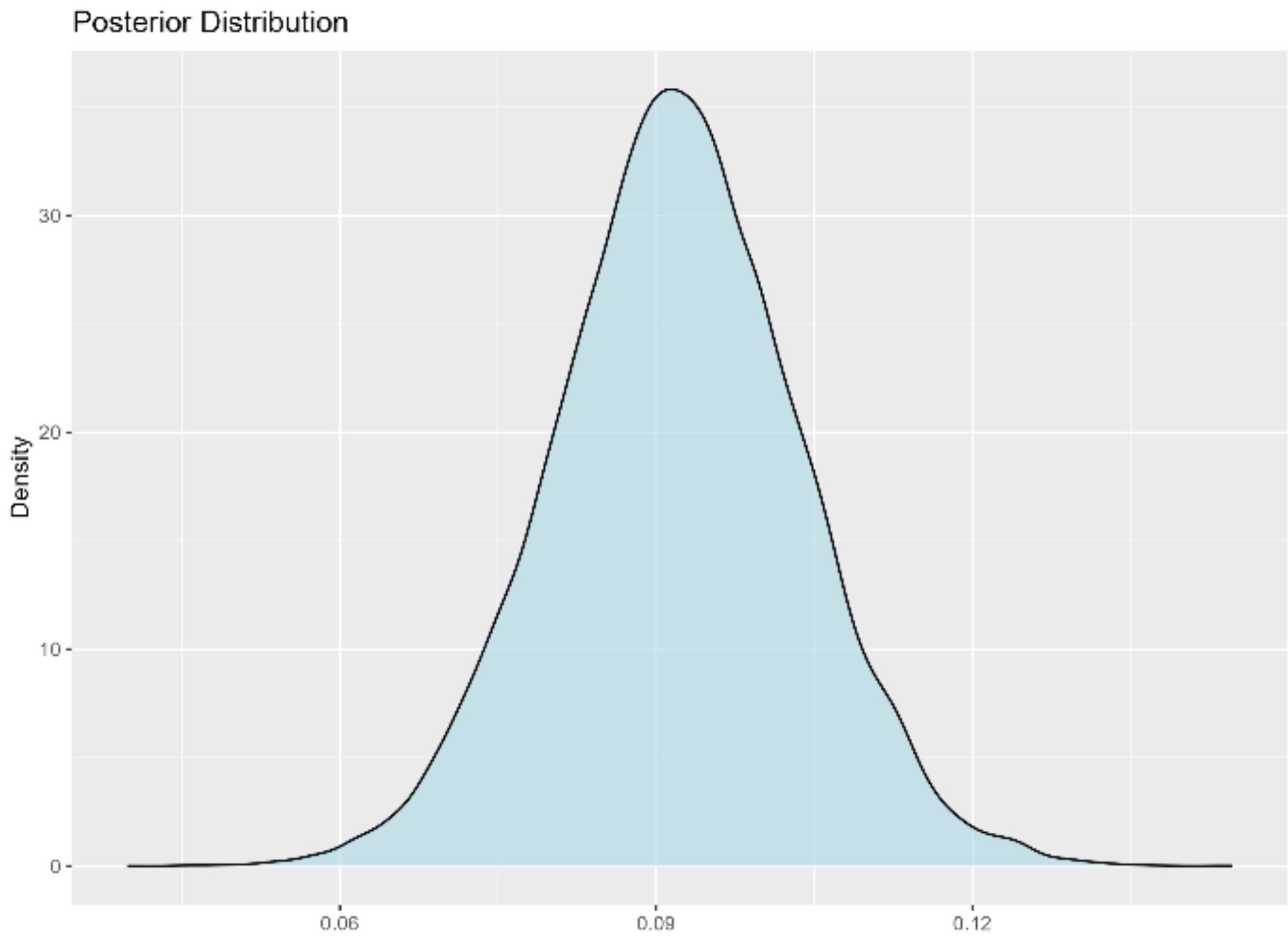

Supplementary Figure 7: Posterior distribution of the automatically-derived planning target volume coverage difference between the daily online adaptive and non-adaptive scenario.

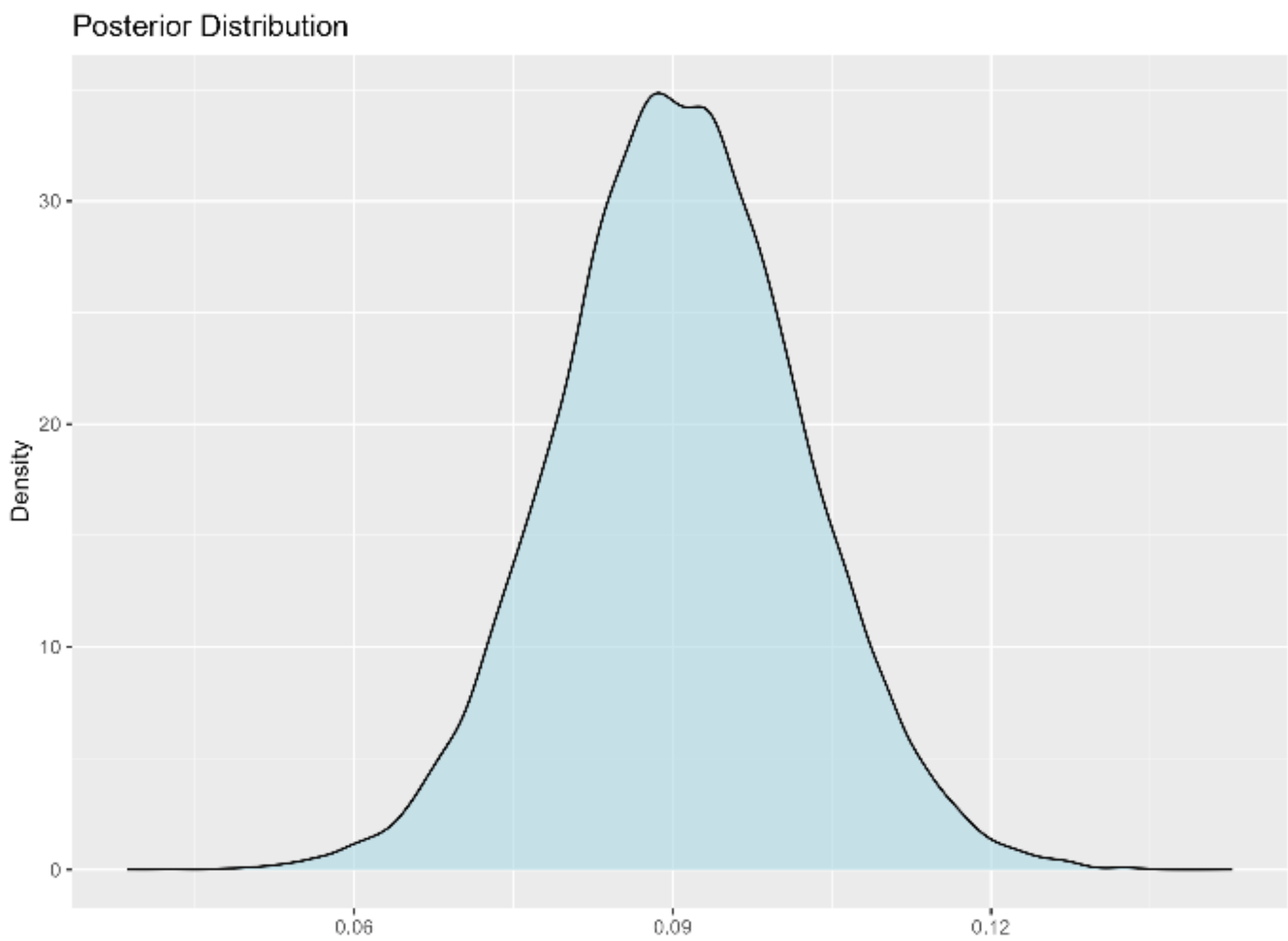

Supplementary Figure 8: Posterior distribution of the automatically-derived planning target volume coverage difference between the daily online adaptive and offline adaptive scenario.

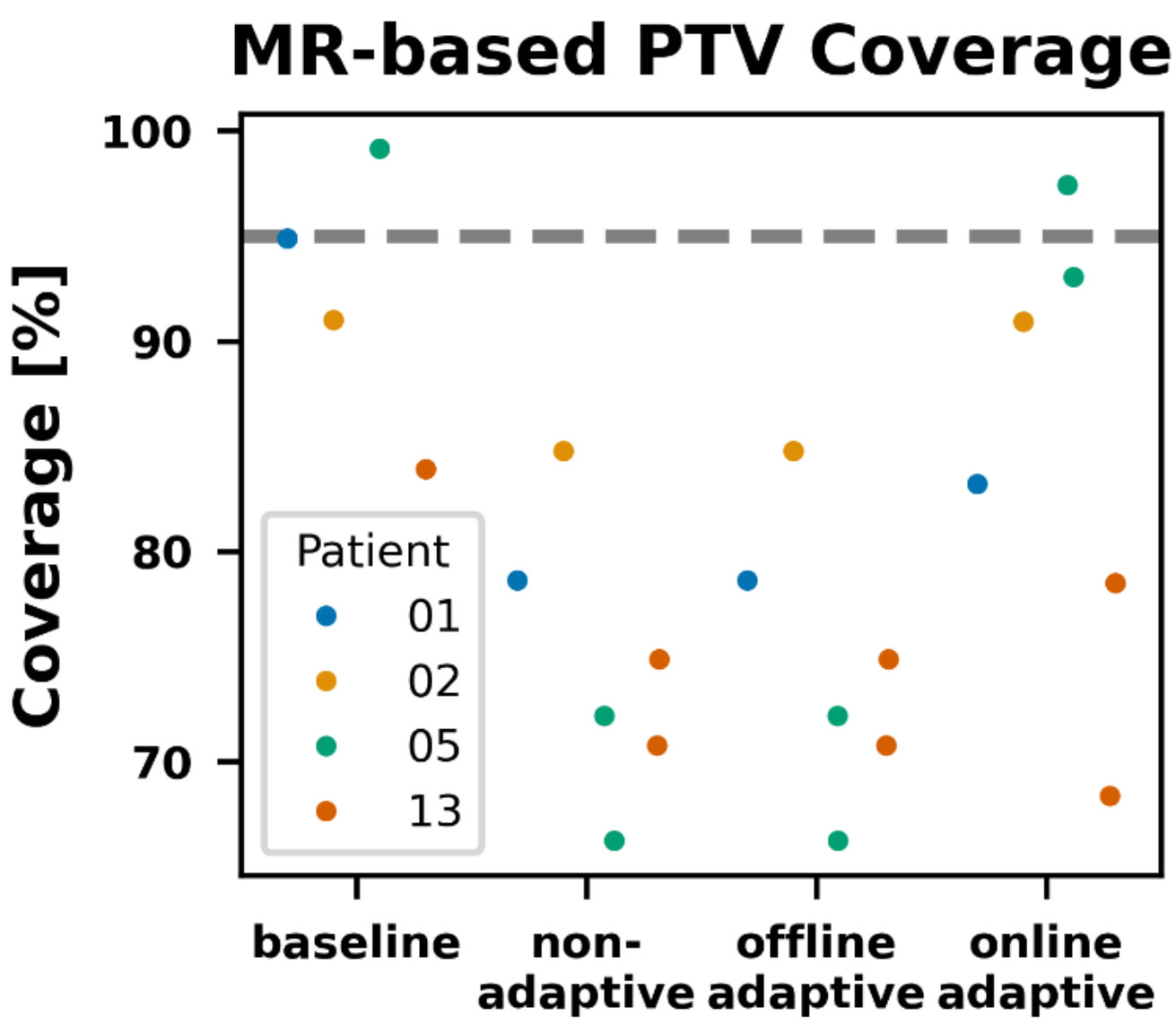

Supplementary Figure 9: Coverage of planning target volume (PTV) based on the MRI-informed gross tumor volume for the different scenarios. The coverage refers to the percentage of the PTV above 95% of the prescribed dose (42 Gy).

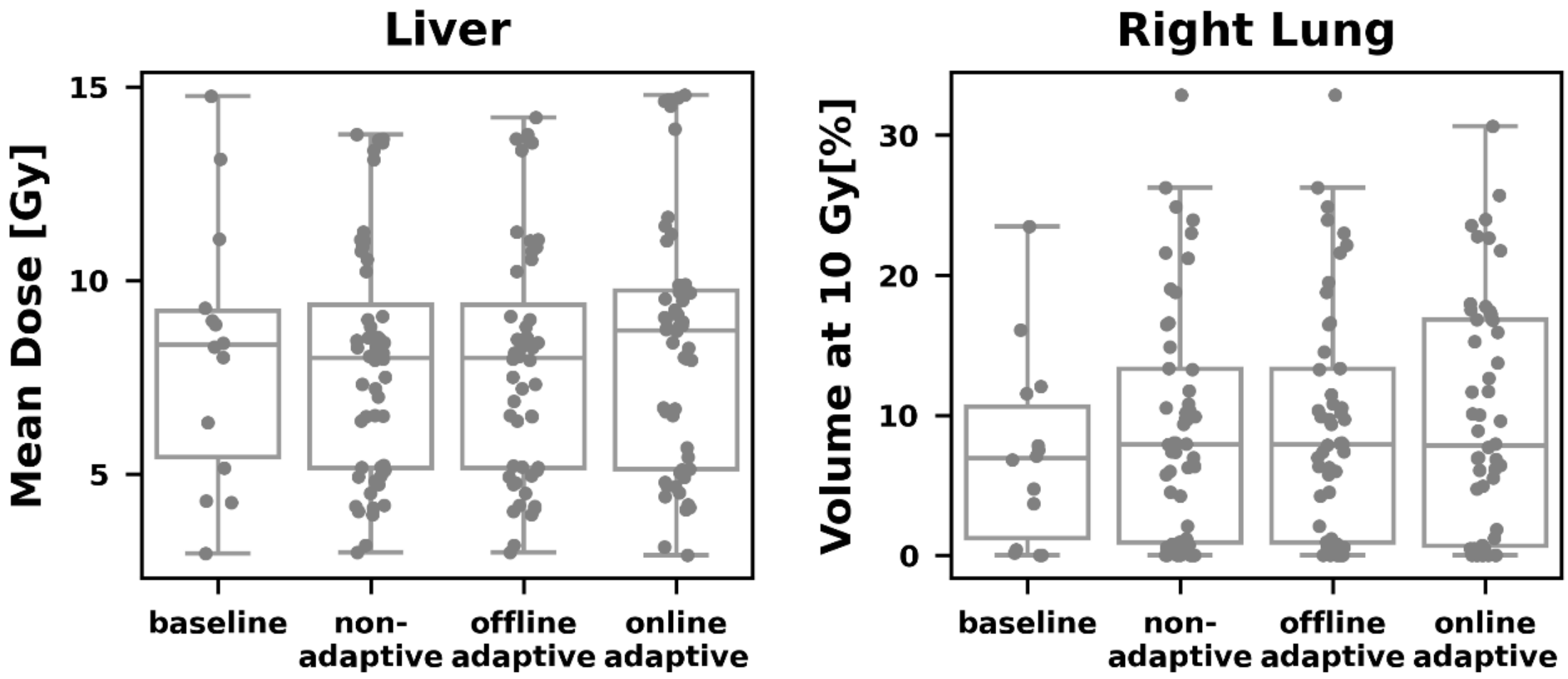

Supplementary Figure 10: Doses inside the liver and lung parenchyma. Left: Mean liver doses. Right: Lung volume at ≥ 10 Gray.

Supplementary Table 1: Results of the Bayesian linear multilevel models for the fully automatic planning target volumes. CI: Bayesian compatibility interval, i.e. respective integral under posterior distribution.

**Parameter**	**Scenario**	**Estimate**	**Error**	**99% CI**
**Intercept**	Baseline	0.95	0.03	0.86 – 1.04
**Coefficients**	Non-adaptive	-0.13	0.03	-0.19 – -0.06
	Offline adaptive	-0.12	0.03	-0.19 – -0.05
	Online adaptive	-0.03	0.03	-0.1 – 0.04
